# Prenatal Use of Sildenafil in Fetal Growth Restriction and Its Effect on Neonatal Tissue Oxygenation—A Retrospective Analysis of Hemodynamic Data From Participants of the Dutch STRIDER Trial

**DOI:** 10.3389/fped.2020.595693

**Published:** 2020-12-03

**Authors:** Fieke Terstappen, Anne E. Richter, A. Titia Lely, Freek E. Hoebeek, Ayten Elvan-Taspinar, Arend F. Bos, Wessel Ganzevoort, Anouk Pels, Petra M. Lemmers, Elisabeth M. W. Kooi

**Affiliations:** ^1^University Medical Center Utrecht, Wilhelmina Children's Hospital, Department of Obstetrics, Utrecht University, Utrecht, Netherlands; ^2^University Medical Center Utrecht, Wilhelmina Children's Hospital and Brain Center, Department for Developmental Origins of Disease, Utrecht University, Utrecht, Netherlands; ^3^University Medical Center Groningen, Beatrix Children's Hospital, Division of Neonatology, University of Groningen, Groningen, Netherlands; ^4^University Medical Center Groningen, Department of Obstetrics, University of Groningen, Groningen, Netherlands; ^5^Amsterdam University Medical Centers, Department of Obstetrics, University of Amsterdam, Amsterdam, Netherlands; ^6^Department of Neonatology, Wilhelmina Children's Hospital, University Medical Center Utrecht, Utrecht University, Utrecht, Netherlands

**Keywords:** fetal growth restriction, Hemodynamics, near-infrared spectroscopy, regional oxygenation, sildenafil

## Abstract

**Objective:** Sildenafil is under investigation as a potential agent to improve uteroplacental perfusion in fetal growth restriction (FGR). However, the STRIDER RCT was halted after interim analysis due to futility and higher rates of persistent pulmonary hypertension and mortality in sildenafil-exposed neonates. This hypothesis-generating study within the Dutch STRIDER trial sought to understand what happened to these neonates by studying their regional tissue oxygen saturation (rSO_2_) within the first 72 h after birth.

**Methods:** Pregnant women with FGR received 25 mg placebo or sildenafil thrice daily within the Dutch STRIDER trial. We retrospectively analyzed the cerebral and renal rSO_2_ monitored with near-infrared spectroscopy (NIRS) in a subset of neonates admitted to two participating neonatal intensive care units, in which NIRS is part of standard care. Secondarily, blood pressure and heart rate were analyzed to aid interpretation. Differences in oxygenation levels and interaction with time (slope) between placebo- and sildenafil-exposed groups were tested using mixed effects analyses with multiple comparisons tests.

**Results:** Cerebral rSO_2_ levels were not different between treatment groups (79 vs. 77%; both *n* = 14) with comparable slopes. Sildenafil-exposed infants (*n* = 5) showed lower renal rSO_2_ than placebo-exposed infants (*n* = 6) during several time intervals on day one and two. At 69–72 h, however, the sildenafil group showed higher renal rSO_2_ than the placebo group. Initially, diastolic blood pressure was higher and heart rate lower in the sildenafil than the placebo group, which changed during day two.

**Conclusions:** Although limited by sample size, our data suggest that prenatal sildenafil alters renal but not cerebral oxygenation in FGR neonates during the first 72 post-natal hours. The observed changes in renal oxygenation could reflect a vasoconstrictive rebound from sildenafil. Similar changes observed in accompanying vital parameters support this hypothesis.

## Introduction

Fetal growth restriction (FGR) increases the risk of perinatal morbidity and mortality (1). FGR is commonly caused by impaired maternal uteroplacental blood flow. To compensate, fetal cardiac output redistributes perfusion toward the brain at the expense of other organs (2). This hemodynamic redistribution remains visible after birth as altered cerebrorenal oxygenation, as demonstrated using near-infrared spectroscopy (NIRS) (3, 4). Although an increase in cerebral oxygenation is intended as protective, it is debated whether this (fully) benefits neurodevelopmental outcome (5, 6). Moreover, redistribution has been associated with impaired cerebral autoregulation (7).

Therapeutic interest in the non-selective phosphodiesterase-5 inhibitor, sildenafil, arose as it may enhance NO-mediated relaxation of the uteroplacental vascular bed (8, 9). Preclinical and small human studies demonstrated improved fetal growth (10). The STRIDER (Sildenafil TheRapy In Dismal prognosis Early-onset fetal growth Restriction) collaboration set up aligned RCTs allocating pregnant women with FGR to either sildenafil or placebo (11). The individual trials demonstrated lack of improved fetal growth and other maternal and perinatal outcomes (12–14). However, the Dutch STRIDER was halted due to futility and higher rates of persistent pulmonary hypertension (PPHN) and mortality in sildenafil-exposed neonates (14). The hemodynamic mechanisms of sildenafil underlying either benefit or harm are unknown.

This study, performed among a subset of neonates from the Dutch STRIDER trial, used a retrospective, hypothesis generating approach in which we sought to understand whether and how prenatal sildenafil affected neonatal hemodynamics, including cerebral and renal tissue oxygenation measured with NIRS (15). This approach can provide critical insights into the pathophysiological mechanisms of sildenafil that might have led to the unanticipated or unintended results of the STRIDER trial.

## Materials and Methods

### Study Population

This hypothesis generating clinical pilot study was retrospectively performed in severe early-onset FGR neonates admitted to the neonatal intensive care unit (NICU) of the University Medical Center of Utrecht (UMCU) and University Medical Center Groningen (UMCG) from November 2015 until August 2018. Their mothers participated in the Dutch STRIDER trial and received either 25 mg sildenafil or placebo thrice daily from randomization until delivery or 32 weeks gestational age, whichever came first (11). The STRIDER study was an RCT recruiting maternal-fetal pairs based on fetal biometric parameters low for gestational age and signs of placental insufficiency. In- and exclusion criteria of this RCT have been reported previously (11). Additionally, we excluded neonates with postnatally evident congenital abnormalities or if postnatal NIRS was not performed due to admission to medium care or another hospital.

The local Medical Ethical Committees of the UMCU (protocol number 15-510/G-C; 14-09-2015) and UMCG (protocol number 2015-252; 12-07-2015) approved participation in the Dutch STRIDER trial. Maternal written informed consent was obtained during pregnancy. In contrast to other centers participating in the Dutch STRIDER trial, standard clinical care at these two NICUs comprises continuous measurements of the regional tissue oxygenation with NIRS and digital storage of this data. These non-invasive measurements are performed by clinical protocol in cerebral (UMCG and UMCU) and splanchnic tissue (UMCG only), and at the discretion of the attending physician also in renal tissue (UMCG and UMCU). Thereby, we were able to study cerebral tissue oxygenation in almost all patients and renal tissue oxygenation in a subset of patients admitted to these NICUs albeit not pre-planned. We did not study splanchnic tissue oxygenation since it was only infrequently and often discontinuously measured within the first days due to a lack of space for an abdominal sensor in these very small infants (*n*_sildenafil_ = 4, *n*_placebo_ = 1). Moreover, neonates born at a gestational age ≥32 weeks without perinatal complications were admitted to the medium care unit, where NIRS measurements are not part of standard care, and are therefore not included in this study.

### Clinical Parameters

Clinical data was derived from the Dutch STRIDER database and patient records. Pulsatility indices of the umbilical and middle cerebral artery from the last complete ultrasound before birth were recorded. The cerebroplacental ratio was calculated dividing the latter by the first, indicating fetal hemodynamic redistribution when below one. Neonatal blood hemoglobin, arterial pCO_2_, and urinary output (per kg body weight) were recorded as daily averages.

### NIRS and Other Hemodynamically Relevant Data

Cerebral and renal regional tissue oxygen saturation (rSO_2_) were continuously measured with NIRS during the first 72 h after birth (INVOS 5100C, Medtronic, Boulder, CO, USA). The cerebral sensor was alternatively placed on the left or right forehead. The UMCU used an adult sensor (SomaSensor SAFB-SM, Medtronic), while the UMCG used a neonatal sensor (OxyAlert CNN NIRSsensor, Medtronic). Due to different algorithms of both sensors, a conversion was used to compare cerebral rSO_2_ data (16): ***rSO***_**2**_**[*neonatalsensor*] = 0**.8481 * *rSO*_**2**_[***adultsensor*** ]**+ 19.11**.

Renal rSO_2_ was measured in a subset of infants using neonatal sensors in both centers wrapped around the posterior-lateral flank right below the costal arch with the tip of the sensor pointing toward the spine (17). The cerebrorenal oxygenation ratio (CRR), based on rSO_2_, was calculated as an indicator of continued hemodynamic redistribution after birth (4).

Simultaneously, arterial oxygen saturation (SaO_2_) and heart rate (HR) were measured using preductal pulse oximetry. The systolic, mean, and diastolic arterial blood pressure (SBP, MABP, and DBP) were invasively measured with an indwelling arterial catheter.

Fractional tissue oxygen extraction (FTOE) was calculated for both brain and kidney using the formula: $FTOE=\frac{{SaO}_{2}-{rSO}_{2}}{{SaO}_{2}}$.

An increased FTOE indicates reduced oxygen supply (perfusion) and/or increased oxygen consumption.

The Pearson correlation coefficients between MABP and cerebral rSO_2_ were determined to estimate the presence or absence of cerebral autoregulation. We calculated the percentage of time per selected 1-h epochs that cerebral autoregulation was impaired as indicated by a correlation coefficient (*r*) > 0.5 (18).

### Processing of Hemodynamic Data

All hemodynamic data were processed using in-house developed software as previously described (Signalbase, UMCU, Utrecht, The Netherlands) (3). Artifacts, defined as physiologically unexplainable changes of at least 30% between two consecutive data points, a lack of variability, or missing data points, were manually removed. A 1-h period of consecutive, high quality rSO_2_ data was selected per 3-h interval and the data of the other parameters were based on this same time period.

Before calculating FTOE or cerebral autoregulation, the two input signals were low pass filtered (-6 dB at 0.01 Hz, implemented by two successive moving average filters of 50 and 36 s) (19). For FTOE, these smoothed signals were resampled to an equal sample rate of 1 Hz to eliminate high-frequency noise in the two signals. For cerebral autoregulation, the smoothed signals were resampled at 0.25 Hz or less, depending on lowest available data rate, before calculating the correlation coefficient over 10 min overlapping periods.

### Statistical Analysis

Statistical analysis was performed with SPSS 25.0 (IBM Corp., Armonk, NY, USA) and GraphPad Prism 8.4.3. (San Diego, CA, USA). Clinical data were tested with an independent Student's *t*-test (mean ± standard deviations), Fisher's exact test [counts (%)], or Mann–Whitney *U* test [median (minimum–maximum)]. Repeated neonatal measurements such as pCO_2_, hemoglobin, and urine output were tested with mixed effects model to compare the daily mean. To test postnatal monitoring and NIRS parameters on group effect (F treatment) and whether the groups behaved differently over time (F interaction treatment × time = F slope), we performed mixed effects model analyses. The model was fitted using Restricted Maximal Likelihood (REML) with individuals as a random effect nested within the groups and compound symmetry as (repeated) covariance type. Greenhouse-Geisser correction was used when sphericity was absent. The individual time points were tested with the uncorrected Fisher's LSD multiple comparisons test. We did not apply *post-hoc* correction to avoid the chance of type 2 error since this was a hypothesis generating study (20–22). Analysis of the data at multiple time points was necessary to fully explore and visualize the effect of sildenafil throughout the whole transition period of these neonates and be able to interpret the data in the context of the findings of the STRIDER trial. Pearson correlation was tested between duration of sildenafil intake and daily averaged NIRS-derived parameters. A two-sided *p*-value below 0.05 was considered significant.

## Results

**Figure 1:**
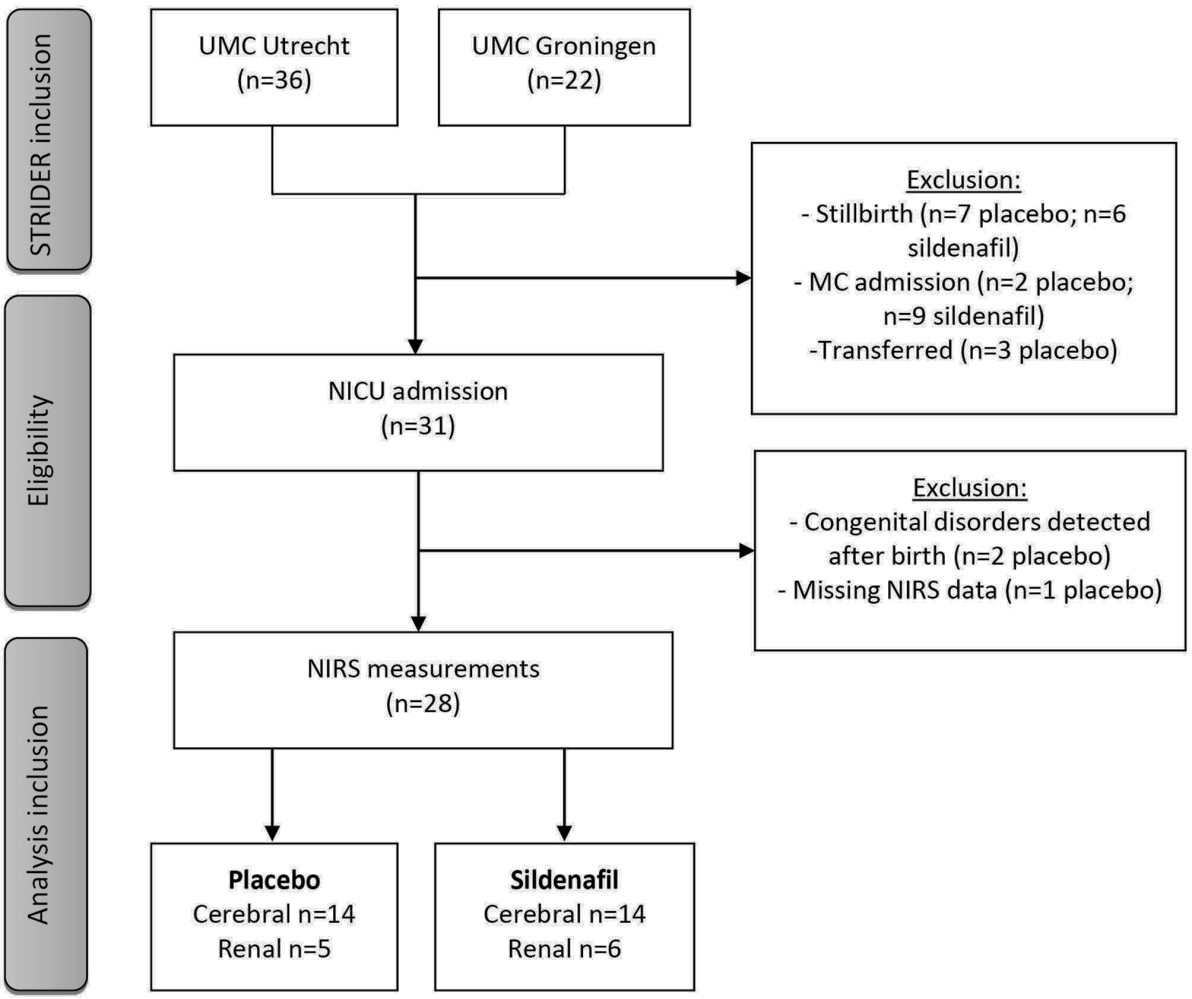
Flow chart of patient inclusion. Neonates were admitted to medium care if they were born at a gestational age ≥32 weeks without perinatal complications. GA, gestational age; NICU, neonatal intensive care unit; MC, medium care; NIRS, near-infrared spectroscopy; UMC, university medical center.

Out of 58 pregnant women participating in the Dutch STRIDER trial, 31 neonates were admitted to the NICU (shown in Figure 1). One patient was excluded due to absent NIRS measurements, and two placebo-exposed patients were excluded because of congenital disorders. In total 14 patients per group were included for analysis, based on cerebral NIRS measurements. Renal NIRS measurements were available in five placebo-exposed vs. six sildenafil-exposed neonates.

### Baseline Characteristics

The median duration of allocated treatment was 15 (0–42) days in the placebo group and 17 (1–44) days in the sildenafil group (Table 1). Severity of FGR and indices of fetal hemodynamic redistribution were comparable at allocation and last ultrasound before birth. There were no differences in birth weight or gestational age. Four placebo-exposed neonates died during hospitalization at a median age of 22 (2–86) days compared to five sildenafil-exposed neonates at a median age of 12 (3–119) days. Infants receiving renal oxygenation measurements were born with a slightly but insignificantly higher birth weight percentile (*p* = 0.09) and developed less commonly bronchopulmonary dysplasia (*p* = 0.04) compared to infants without renal measurements. There were no differences in gestational age, hemodynamic significant persistent ductus arteriosus, necrotizing enterocolitis, PPHN, or mortality.

**Table 1:** Baseline characteristics.

	**Placebo(*n* = 14)**	**Sildenafil(*n* = 14)**	***P*-value**
**Maternal characteristics during pregnancy**			
Age (years)	31 (28–43)	31 (24–43)	0.49
PEH	3 (21)	1 (7)	0.60
PIH	5 (36)	5 (36)	1.00
PE	8 (57)	6 (43)	0.71
HELLP	3 (21)	1 (7)	0.60
PPROM	0 (0)	1 (7)	1.00
Smoking	2 (14)	2 (14)	1.00
**Prenatal administration of allocated drug**			
GA at start (weeks)	25.4 ± 1.2^a^	25.4 ± 1.3	0.98
GA at stop (weeks)	28.1 ± 1.4^a^	27.9 ± 2.1	0.77
Duration (days)	17.4 ± 14.9	17.4 ± 13.3	1.00
**Maternal medication during pregnancy**			
Labetalol	9 (64)	8 (57)	1.00
Methyldopa	7 (50)	5 (36)	0.70
Nifedipine	5 (36)	4 (29)	1.00
Aspirin	3 (21)	1 (7)	0.60
MgSO_4_	7 (50)	8 (57)	0.70
**Prenatal ultrasound at allocation**			
GA at ultrasound (weeks)	25.1 ± 1.5	25.1 ± 1.7	0.88
EFW < p3	10 (71)	12 (92)	0.33
HC < p3	8 (62)	10 (77)	0.67
AC < p3	10 (71)	10 (77)	1.00
HC/AC ratio	1.24 ± 0.06	1.21 ± 0.10	0.33
UA PI	1.77 ± 0.60	2.11 ± 0.72	0.19
MCA PI	1.44 (0.88–1.98)	1.34 (0.90–5.27)	0.82
CPR < 1 (hemodynamic redistribution)	9 (69)	10 (77)	1.00
**Last complete prenatal Doppler ultrasound**			
UA PI	2.12 (0.78–2.52)^a^	2.08 (1.06–3.52)^a^	0.95
MCA PI	1.51 (1.19–2.10)^a^	1.30 (0.88–2.42)^a^	0.10
CPR < 1 (hemodynamic redistribution)	10 (91)^a^	8 (62)^a^	0.17
**Delivery**			
Cesarean section	14 (100)	12 (86)	0.48
Apgar 5	8 (5–9)	7 (6–9)^a^	0.70
**Neonatal characteristics**			
Male (%)	10 (71)	8 (57)	0.70
GA at birth (weeks)	28.1 ± 1.3	28.4 ± 2.6	0.72
Birth weight (g)	688 (490–1170)	620 (440–1140)	0.32
Birth weight (p)			0.88
*<p3*	9 (64)	10 (71)	
*p3-10*	2 (14)	2 (14)	
*>p10*	3 (21)	2 (14)	
HC (cm)	23.0 (21.0–28.4)	22.5 (20.0–25.0)	0.10
HC (p)			0.43
*<p3*	5 (36)	8 (57)	
*p3-10*	3 (21)	3 (21)	
*>p10*	6 (42)	3 (21)	
**Neonatal morbidity and mortality during NICU admission**			
IVH	3 (21)	5 (36)	0.68
NEC	2 (14)	2 (14)	1.00
Early onset sepsis	1 (7)	1 (7)	1.00
Late onset sepsis	4 (29)	3 (21)	1.00
PPHN	1 (7)	3 (21)	0.60
IRDS	12 (86)	9 (64)	0.39
BPD	6 (43)	4 (29)	0.70
hsPDA	7 (50)	6 (43)	1.00
Mechanical ventilation	14 (100)	13 (93)	1.00
Neonatal death prior to discharge	4 (29)	5 (36)	1.00
*Age at death (days)*	33 ± 39	43 ± 52	0.77
NICU admission (days) in survivors	48 (33–117)	60 (21–83)	0.54
**Neonatal medication during first 3 days**			
Postnatal steroids	1 (7)	3 (21)	0.60
hsPDA treatment	0 (0)	1 (7)	1.00
Surfactant	10 (71)	8 (57)	0.70
Inotropes	3 (23)^a^	3 (23)^a^	1.00
Caffeine	13 (93)	12 (86)	1.00
NO inhalation	0 (0)	1 (8)^a^	0.48
**Neonatal laboratory values during first 3 days**			
Hb (mmol/L)			0.64
*Day 1*	10.3 ± 1.2	11.1 ± 1.4	0.16
*Day 2*	9.3 ± 1.3	10.2 ± 1.3	0.06
*Day 3*	9.1 ± 1.0^a^	9.6 ± 1.2^a^	0.26
pCO_2_ (mmHg)			0.42
*Day 1*	41.0 ± 8.7	46.8 ± 7.6	0.08
*Day 2*	44.6 ± 8.2	43.3 ± 8.2^a^	0.34
*Day 3*	45.8 ± 9.1^a^	41.4 ± 5.1^a^	0.36
Urine production (ml/kg body weight)			0.97
*Day 1*	88.2 ± 39.8	104.5 ± 50.9^a^	0.36
*Day 2*	87.6 ± 35.1	108.0 ± 37.9^a^	0.16
*Day 3*	79.4 ± 24.9^a^	99.6 ± 51.6^a^	0.22

*Data are expressed as mean ± SD, n(%) or median (minimum-maximum)*.

a *due to missing data percentages are calculated based on the number of observations/measurements within the treatment group with 11 being the lowest number of patients in a group (maximum missing data of 21%). Prophylactic low-dose aspirin (100 mg) before 16 weeks' gestation was registered. Percentiles of prenatal biometry were determined using the perinatology biometry calculator (http://www.perinatology.com/calculators/biometry.htm). Percentiles for weight and head circumference at birth were determined with Intergrowth-twenty first (23). IVH was defined as grade 3 or higher, NEC as Bell stage 2a or higher, BPD was defined as need of oxygen ≥21% for ≥28 days postnatal age or at discharge. AC, abdominal circumference; CPR, cerebroplacental ratio; EFW, estimated fetal weight; FL, femur length; GA, gestational age; HC, head circumference; HELLP, Hemolysis, Elevated Liver enzymes, and Low Platelet syndrome; hsPDA, treatment-requiring patent ductus arteriosus; IRDS, infantile respiratory distress syndrome; IVH, interventricular hemorrhage; MCA, middle cerebral artery; NEC, necrotizing enterocolitis; NICU, neonatal intensive care unit; p, percentile; PE, pre-eclampsia; PEH, pre-existent hypertension; PI, pulsatility index; PIH, pregnancy induced hypertension; PPHN, persistent pulmonary hypertension of the neonate; PPROM, preterm premature rupture of membranes; UA, umbilical artery*.

### Cerebral and Renal Tissue Oxygenation

**Figure 2:**
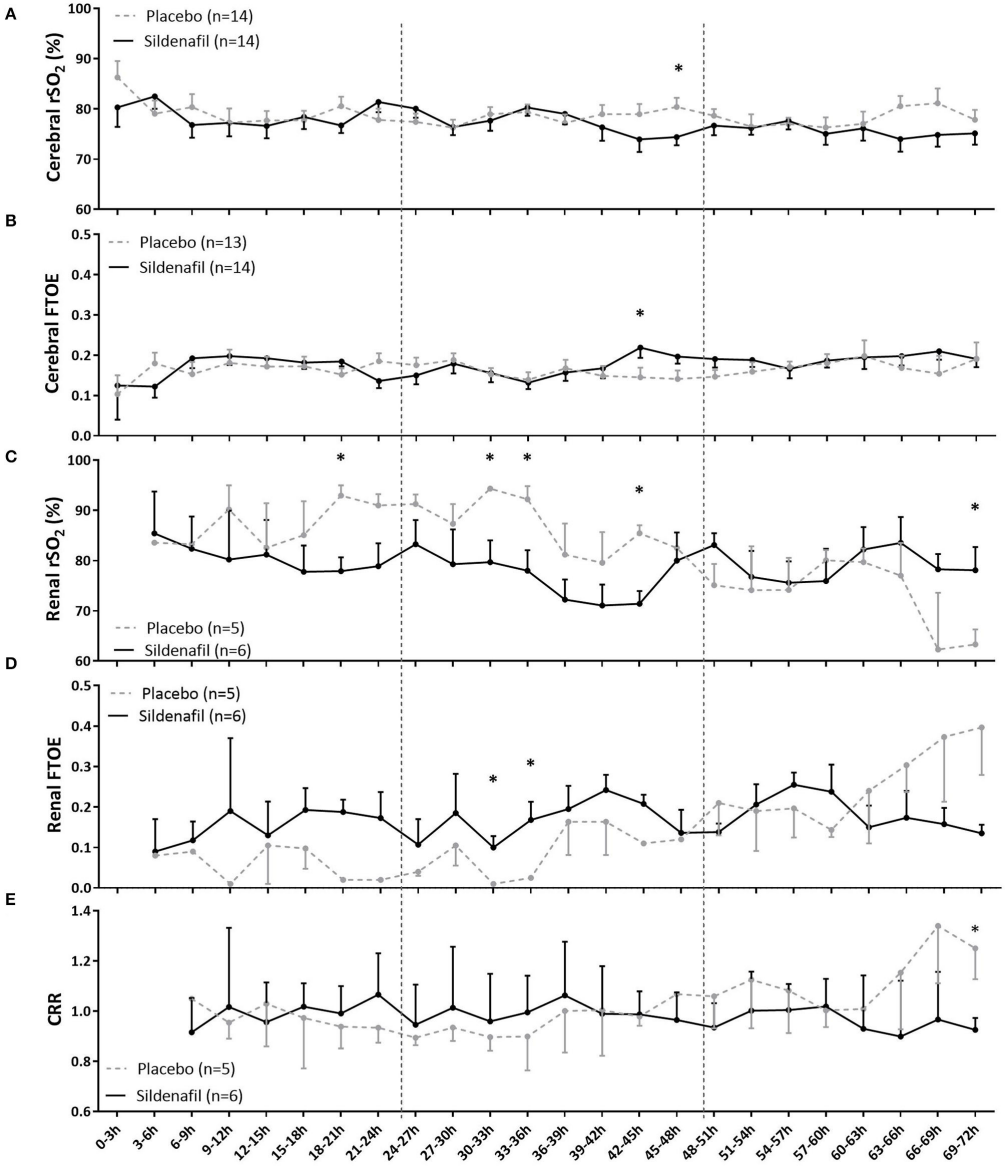
The effect of prenatal sildenafil on tissue oxygenation during the first 3 days after birth in severe early-onset fetal growth restricted neonates. The panels show the **(A)** cerebral regional oxygen saturation (rSO_2_), **(B)** cerebral fractional tissue oxygen extraction (FTOE), **(C)** renal rSO_2_, **(D)** renal FTOE, and **(E)** cerebrorenal ratio (CRR) between cerebral and renal rSO_2_. Data are expressed as mean±SEM. The asterisks indicate which time intervals differed between groups at *p* < 0.05 according to uncorrected multiple comparisons tests. Vertical dotted lines indicate days after birth.

Overall, the level and slope of cerebral rSO_2_ and FTOE were not different between neonates in the sildenafil and placebo arm during the first 72 postnatal hours (Supplementary Table 1). Only at one time interval, cerebral rSO_2_ was lower (at 45–48 h, Figure 2A) and cerebral FTOE higher (at 42–45 h, Figure 2B) in sildenafil-exposed infants. Sub-analysis including only infants with both cerebral and renal measurements, as there may have been a systematic difference between neonates receiving both cerebral and renal measurements and all neonates, did not relevantly alter our results regarding cerebral rSO_2_ and autoregulation (Supplementary Table 2). Cerebral FTOE levels in the sildenafil-exposed group were similar compared to our original results, but slightly and just significantly lower in the placebo-exposed group (F_treatment_: *p* = 0.047, Supplementary Table 2).

Slope and overall levels of renal rSO2 were also not different between groups (Supplementary Table 1). However, sildenafil-exposed infants had lower renal rSO2 during several time intervals on postnatal day one (18–21 h) and two (30–33 h, 33–36 h, and 42–45 h), but higher renal rSO2 at the end of postnatal day three (69–72 h) (Figure 2C). While there was no overall treatment effect on renal FTOE, the sildenafil group showed higher FTOE at two time intervals during postnatal day two (30–36 h) (Figure 2D).

CRR of both groups were comparable in overall level and slope (Supplementary Table 1). Only during 69–72 h, CRR was significantly lower in the sildenafil group (Figure 2E).

The duration of sildenafil use did not correlate with cerebral rSO_2_ or FTOE or the CRR. A longer use of sildenafil, however, correlated significantly with a higher renal FTOE on postnatal days two and three (both *r* = 0.92, *p* < 0.05) (Table 2).

**Table 2:** Pearson correlation coefficient (*r*) between maternal sildenafil intake and the cerebral and renal NIRS-derived parameters in early-onset fetal growth restriction.

**Postnatal parameter**	***r*_**day 1**_ (*n*)**	***r*_**day 2**_ (*n*)**	***r*_**day 3**_ (*n*)**
Cerebral rSO_2_	−0.393 (12)	−0.001 (14)	−0.101 (12)
Renal rSO_2_	−0.461 (5)	−0.792 (6)	−0.269 (6)
CRR	−0.299 (5)	−0.228 (6)	−0.231 (6)
Cerebral FTOE	0.320 (11)	0.269 (13)	0.292 (12)
Renal FTOE	0.679 (5)	0.915 (5)^*^	0.915 (5)^*^

* *P < 0.05. CPR, cerebroplacental ratio; CRR, cerebrorenal rSO_2_ ratio; FTOE, fractional tissue oxygen extraction; rSO_2_, regional oxygen saturation*.

### Arterial Oxygen Saturation, Heart Rate and Blood Pressure

**Figure 3:**
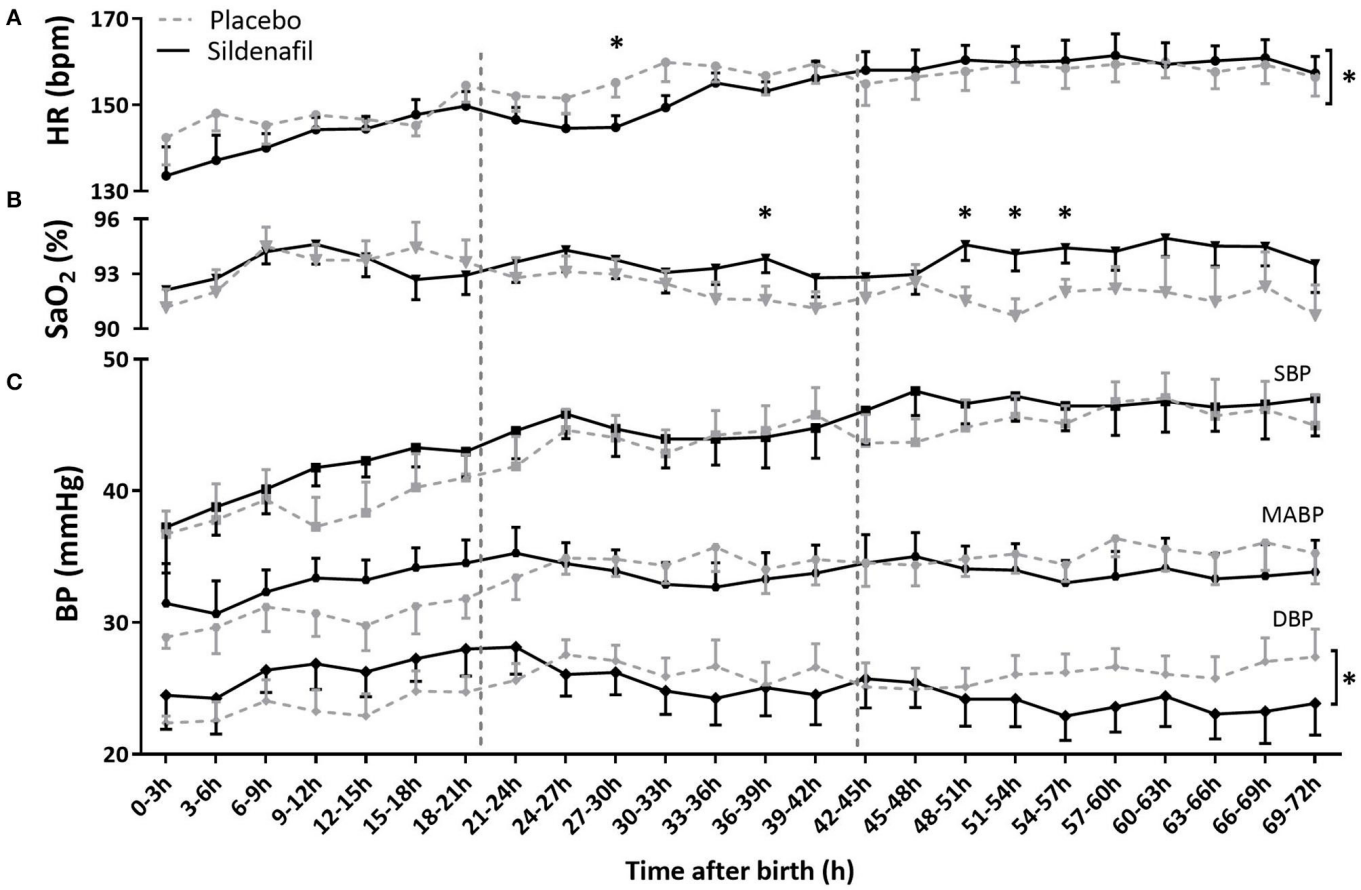
The effect of sildenafil on **(A)** heart rate (HR), **(B)** arterial saturation (SaO_2_) and **(C)** blood pressure (BP) during the first 3 days after birth in severe early-onset fetal growth restricted neonates. HR and BP are shown in *n* = 12 placebo- vs. *n* = 11 sildenafil-exposed neonates, and SaO_2_ in *n* = 13 placebo- vs. *n* = 14 sildenafil-exposed neonates. Data are expressed as mean ± SEM. The asterisks indicate which time intervals differed between groups at *p* < 0.05 according to uncorrected multiple comparisons tests. The asterisks behind brackets at the end of the graph indicate a difference in slope at *p* < 0.5 between groups tested with mixed model analysis.

Although there was no overall difference in DBP or HR level either, DBP and HR behaved differently over time with significantly steeper slopes in the sildenafil group (Supplementary Table 1; Figures 3A,C). While infants in the sildenafil group started at a lower HR (with a significant difference at 27–39 h) and a higher DBP (statistically insignificant at individual time points), HR was equal or slightly higher and DBP was lower on day three in these infants (without statistically significant differences at individual time points). SBP and MABP were not different in level and slope between groups (Supplementary Table 1; Figure 3C).

While SaO_2_ levels were not different between groups on day 1 after birth, sildenafil-exposed infants showed a higher SaO_2_ at four time points at the end of day 2 and during day 3. However, slope and overall level were not different (Supplementary Table 1; Figure 3B).

### Cerebral Autoregulation

**Figure 4:**
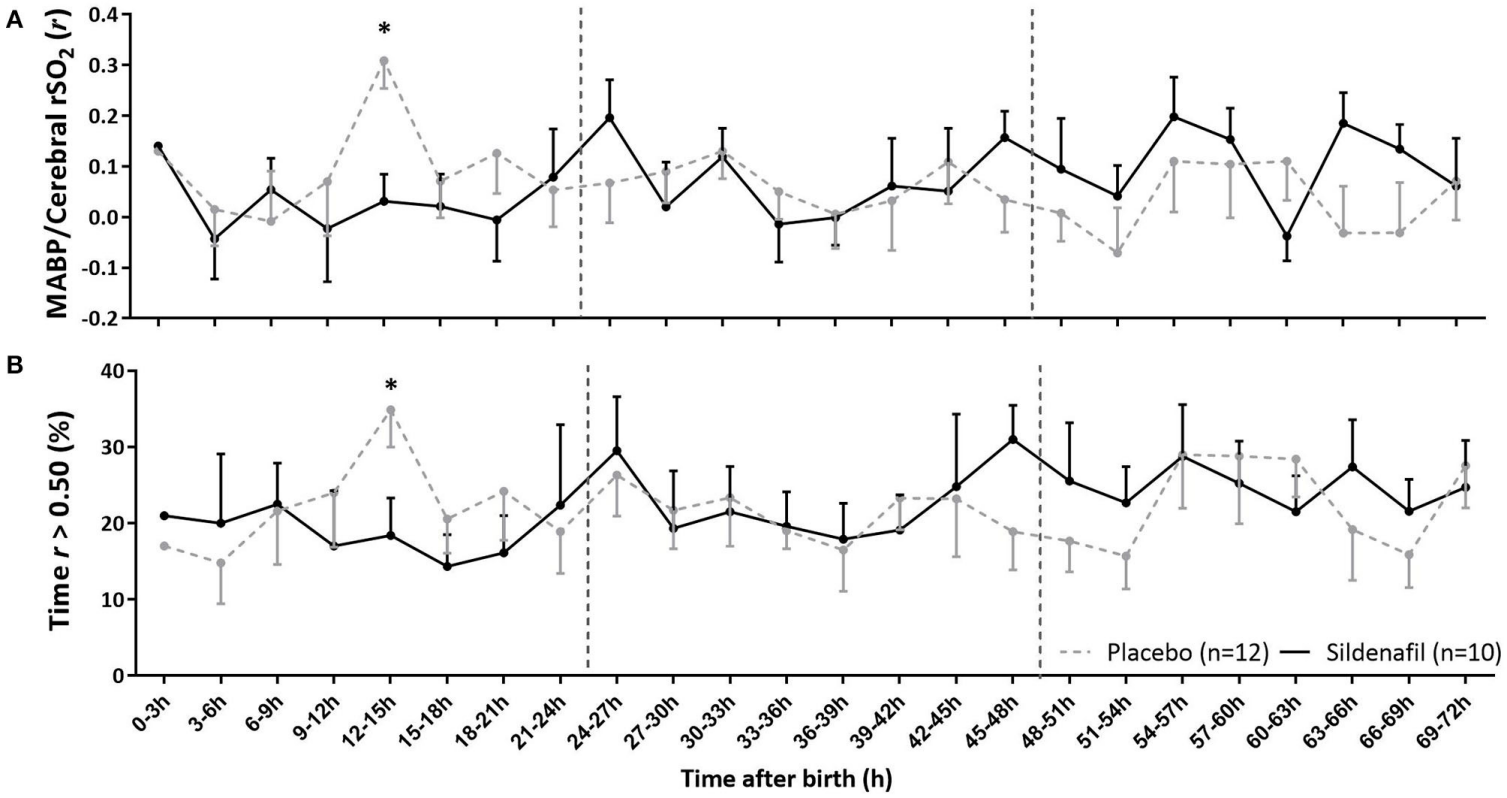
The effect of sildenafil on cerebral autoregulation during the first 3 days after birth in severe early-onset fetal growth restricted neonates. The panels show **(A)** the correlation coefficient (*r*) between MABP and cerebral rSO_2_ and **(B)** the percentage time that *r* is >0.50, indicating impaired cerebral autoregulation. Data are expressed as mean ± SEM. The asterisk indicate which time intervals differed between groups at *p* < 0.05 according to uncorrected multiple comparisons tests. Vertical dotted lines indicate days after birth.

Correlation coefficients between MABP and cerebral rSO_2_ and the percentage of time of impaired cerebral autoregulation were not different in level and slope between groups (Table 2). Only at 12–15 h sildenafil-exposed infants showed a lower mean correlation coefficient (Figure 4A) and less exposure to impaired cerebral autoregulation (Figure 4B) than placebo-exposed infants.

## Discussion

This hypothesis generating retrospective pilot study examined whether and how prenatal sildenafil may affect cerebral and renal tissue oxygenation in severe early-onset FGR during the first postnatal 72 h. We observed no overall difference in (i) cerebral rSO_2_, FTOE, or autoregulation, (ii) renal rSO_2_ or FTOE, and (iii) cerebrorenal oxygenation ratio between the groups. However, while cerebral rSO2, FTOE and autoregulation were also largely similar regarding individual time intervals, sildenafil-exposed infants appeared to have lower renal rSO_2_ and higher renal FTOE during several time intervals on the first two postnatal days, suggesting either decreased renal perfusion or increased renal oxygen consumption. At the end of postnatal day three renal rSO_2_ was higher. Moreover, longer maternal treatment with sildenafil correlated with higher renal FTOE on day two and three. These findings suggest that sildenafil may directly affect neonatal renal oxygenation and perfusion, but less so cerebral oxygenation or perfusion.

With regards to the brain, a largely unaffected oxygenation and autoregulation by sildenafil may be reassuring, given the systemic and local vasodilatory potential of sildenafil. However, it also demonstrates no clear improvement of cerebral perfusion in a population in whom the cerebral oxygenation and autoregulation is frequently disturbed. Likewise, a lack of difference in cerebrorenal oxygenation status combined with absence of any observable increase in cerebroplacental ratio or birth weight, suggests that sildenafil did not improve placental function and fetal hemodynamics in this cohort. This is in line with the findings of the individual STRIDER studies, which reported no improvement of uteroplacental perfusion, fetal hemodynamics and growth after sildenafil treatment (12–14). While this lack of effect questions the general use of sildenafil, it may also relate to insufficient power or underdosage of sildenafil, since in animal studies, higher sildenafil dosages resulted in greater effects on fetal growth (10).

Our data suggests a lower renal oxygenation on day one and two, but higher renal oxygenation on day three in sildenafil-exposed compared to placebo-exposed infants, which may relate to direct or indirect changes in renal perfusion. Sildenafil has been demonstrated to directly preserve renal cGMP-levels, enhance NO signaling, and thereby improve renovascular relaxation (24–26). Unaltered cerebral oxygenation further supports enhanced renovascular perfusion through a direct effect of sildenafil rather than improved placental function and fetal perfusion. Although a difference in rSO_2_ of 10% between groups is likely to be clinically relevant, it remains speculative whether the observed changes in renal oxygenation may be beneficial or harmful for the neonate. Of note, daily urine output was higher following sildenafil, which may relate to a vasodilatory effect in utero, suggested by a high renal rSO_2_ and low renal FTOE on postnatal day three. Although statistically non-significant, a 20 ml higher daily urinary output in FGR infants, who weigh <1 kg and commonly present with reduced urine production, may be clinically relevant (27, 28). The interpretation of this finding may be an inability to retain water, but it may also be a sign of better kidney function (29).

Secondary analysis of accompanying vital parameters revealed similar patterns as renal oxygenation during the 72 h after birth, especially for DBP and HR. Since, generally, sildenafil lowers BP without directly affecting HR, a higher BP as observed on day one may reflect a rebound effect following sudden drug withdrawal at birth, with a compensatory change in HR (30, 31). Given the similar pattern of renal oxygenation within the first 72 h, these changes may reflect direct vasodilatory effects of sildenafil on the renal vasculature, which is visible by the end of day three following an initial vasoconstrictive rebound up until day two. Similarly, sildenafil may affect systemic BP, although altered BP could also relate to altered renal function.

This speculative vasoconstrictive rebound following sildenafil withdrawal may be similarly responsible for the higher incidence of PPHN in infants exposed to sildenafil within the Dutch STRIDER trial. It might have warranted further research into a potential benefit of postnatal sildenafil continuation to slowly wean infants, were it not that beneficial effects on fetal growth were not observed in the main trial (14). The exact mechanism behind the vasoconstrictive rebound remains speculative, although downregulation of endogenous NO may play a role (32). Moreover, its timing of onset and duration may be difficult to predict. While sildenafil has a half-life of 3–4 h in adults, placental transfer of sildenafil has shown to be increased in preeclampsia and clearance may be reduced in pregnancy and preterm infants (33–36). Indeed, renal FTOE on day two and three but not on day one positively correlated with duration of maternal sildenafil intake, suggesting that the sustained sildenafil might result in a stronger and longer rebound effect. Prolonged sildenafil intake may therefore delay postnatal clearance of sildenafil and prolong hemodynamic rebound. However, we do not have not have pharmacokinetic or relevant metabolite data to support this.

This study utilized both original data from the Dutch STRIDER trial and data from routine clinical care. Because the effects of prenatal sildenafil on neonatal hemodynamics have not been investigated before, the results of this study are unique and contribute to a better understanding of the Dutch STRIDER results. Moreover, being able to study a population subset of a well-executed randomized controlled trial with strict inclusion criteria, our presented hemodynamic data were limited to infants truly affected by placental insufficiency. However, we acknowledge some study limitations. First, although this was a hypothesis generating and not hypothesis testing study, we are aware that the small number of included neonates limited power to detect significant differences, in particular concerning renal oxygenation. Unfortunately, NIRS and digital storage of high frequency data are not routinely implemented in other Dutch centers, explaining why we could only retrospectively examine data collected at two of the participating centers. Second, we analyzed multiple time points and variables without correcting for multiple testing, which may have increased the chance of type 1 error. This was, however, necessary to carefully examine the holistic effect of sildenafil on the neonatal hemodynamics throughout the whole transitional period and fully understand a possible lack of effect of sildenafil and any proposed increase of PPHN and mortality by this drug. Moreover, since this study set out to generate a hypothesis on what happened to the neonates of the STRIDER trial, we actively decided to refrain from a correction of multiple testing to decrease the chance of type 2 error. Third, the small sample limited the incorporation of other influencing factors such as hsPDA, maternal antihypertensive medication, caffeine, or inotropes, which—even though they were similarly distributed among both randomized groups—may be sustained by or have an additive effect with sildenafil. Fourth, it would have been methodologically more valid if both included centers used the same type of sensors to measure cerebral oxygenation. However, the formula converting algorithms used by the adult to those of the neonatal sensor was developed in a large group of neonates with a broad range of gestational ages at birth, so we believe its influence on our findings being negligible (16). Instead, it may be worthwhile to emphasize that neither centrum performed an ultrasound to confirm correct placing of the renal NIRS sensor. Fifth, renal oxygenation was only measured on indication, studying a group of infants potentially more severely ill than the cerebral NIRS group. This may have introduced a systematic difference between neonates receiving both cerebral and renal measurements and all neonates, potentially explaining why we found an effect in renal NIRS but not cerebral NIRS. Subanalyses of cerebral NIRS in only those neonates with both cerebral and renal NIRS measurements can neither support nor exclude this possibility. Finally, although the rate of stillbirths was similar between the sildenafil and placebo group, relatively more sildenafil-exposed neonates were admitted to the medium care unit and excluded from analyses. Although the characteristics of admitted neonates were similar, the resultant potential selection bias may underestimate beneficial effects of sildenafil.

In conclusion, the unique but also limited data of this hypothesis generating study suggest that prenatally administered sildenafil did not affect cerebral oxygenation or autoregulation in this subset of FGR neonates from the Dutch STRIDER. This may be reassuring given the systemic and cerebral vasodilatory potential of sildenafil and the potentially adverse outcomes observed in neonates participating in the Dutch STRIDER trial. In combination with an unaltered cerebrorenal oxygenation ratio, however, it also suggests little improvement of placental function and subsequent fetal hemodynamics. This may relate to either futility or inadequate dosage of sildenafil, small sample size, or the potential exclusion of neonates not requiring intensive care due to better placental function. Yet, sildenafil may influence renal perfusion as we observed a lower renal rSO_2_ and higher renal FTOE in the sildenafil group compared to the placebo group within the first 48 h after birth with an opposite finding on day three. Similar patterns of accompanying vital parameters generate the hypothesis that sildenafil has a direct vasodilatory effect on the renal vasculature, masked by an initial vasoconstrictive rebound-phenomenon, which may have contributed to higher PPHN and mortality rates within the Dutch STRIDER trial. Moreover, the strength of this phenomenon may positively correlate with the duration of drug intake. We recommend future (most likely animal) studies to test this hypothesis, closely monitoring pharmacokinetic alongside physiological changes. Furthermore, future (follow-up) studies should involve assessment of renal function and neurocognitive outcome.

## Data Availability Statement

The original contributions presented in the study are included in the article/Supplementary Materials, further inquiries can be directed to the corresponding author/s.

## Ethics Statement

The studies involving human participants were reviewed and approved by Medical Ethical Committees of the university medical center Utrecht and the University Medical Center Groningen. Written informed consent to participate in this study was provided by the participants' legal guardian/next of kin.

## Author Contributions

FT contributed to the study design, collected, analysed, and interpreted the data, and drafted the initial manuscript. AR contributed to the study design, collected, and interpreted the data, and drafted the initial manuscript. AL conceptualized the study design, interpreted the data, and critically reviewed the manuscript for intellectual content. FH, AE-T, AB, and WG contributed to the interpretation of data and critically reviewed and revised the manuscript for intellectual content. AP contributed to the acquisition and interpretation of data, and critically reviewed and revised the manuscript for intellectual content. PL and EK conceptualized and designed the study, supervised data collection and interpretation, and critically reviewed and revised the manuscript for intellectual content. All authors approved the final manuscript as submitted and agree to be accountable for all aspects of the work.

## Conflict of Interest

The authors declare that the research was conducted in the absence of any commercial or financial relationships that could be construed as a potential conflict of interest.
