# Supplementary material for: Prenatal Use of Sildenafil in Fetal Growth Restriction and Its Effect on Neonatal Tissue Oxygenation—A Retrospective Analysis of Hemodynamic Data From Participants of the Dutch STRIDER Trial
Source: Front Pediatr. 2020 Dec 3;8:595693. doi: 10.3389/fped.2020.595693 (PMC7744464; doi:10.3389/fped.2020.595693)
Supplement: Supplementary file 1 [file Data_Sheet_1.docx]

Supplementary Material

# Supplementary Tables

**Table S1.** The effect of prenatal sildenafil versus placebo on neonatal NIRS-derived and **vital**parameters and cerebral autoregulation in severeearly-onset fetal growth restriction.

| **Parameter** | **Mean** | | | **Difference between means (95% CI)** | **SE of difference** | **Treatment** | | **Slope** | | |  |
| --- | --- | --- | --- | --- | --- | --- | --- | --- | --- | --- | --- |
|  | **Placebo** | **Sildenafil** | |  |  | **F (dfn; dfd)** | **p** | **F (dfn; dfd)** | **p** |  |  |
| **NIRS-derived parameters** | | | | | | | | | | | |
| Cerebral rSO_2_ (%) | 78.50 | | 76.88 | 1.61 (-1.74; 4.96) | 1.63 | 0.98 (1; 26) | 0.33 | 1.18 (23; 488) | 0.26 |  |  |
| Renal rSO_2_ (%) | 81.13 | | 79.36 | 1.778 (-8.406; 11.96) | 4.50 | 0.16 (1; 9) | 0.70 | 1.26 (22; 116) | 0.22 |  |  |
| CRR | 1.04 | | 0.98 | 0.06 (-0.08; 0.20) | 0.06 | 0.95 (1; 9) | 0.36 | 1.54 (21; 109) | 0.08 |  |  |
| Cerebral FTOE | 0.16 | | 0.18 | -0.02 (-0.05; 0.02) | 0.02 | 0.99 (1; 25) | 0.33 | 0.92 (23; 416) | 0.58 |  |  |
| Renal FTOE | 0.13 | | 0.17 | -0.04 (-0.15; 0.07) | 0.04 | 0.64 (1; 9) | 0.44 | 1.40 (22; 87) | 0.14 |  |  |
| **Vital parameters** | | | | | | | | | | | |
| HR (bpm) | 155 | | 153 | 2 (-6; 11) | 4 | 0.28 (1; 21) | 0.60 | 2.01 (23; 394) | 0.004 |  |  |
| SBP (mmHg) | 43 | | 45 | -2 (-6; 3) | 2 | 0.68 (1; 22) | 0.42 | 0.84 (23; 402) | 0.68 |  |  |
| DBP (mmHg) | 26 | | 26 | -0.001 (-4.1; 4.1) | 2 | <0.001 (1; 22) | 1.00 | 1.59 (23; 402) | 0.04 |  |  |
| MABP (mmHg) | 34 | | 34 | -0.1 (-4.2; 3.9) | 2 | 0.005 (1; 21) | 0.95 | 1.14 (23; 379) | 0.29 |  |  |
| SaO_2_ (%) | 92 | | 93 | -1 (-3; 1) | 1 | 1.49 (1; 25) | 0.23 | 1.19 (23; 504) | 0.25 |  |  |
| **Cerebral autoregulation** | | | | | | | | | | | |
| MABP/cerebral rSO_2_correlation coefficient (*r*) | 0.08 | | 0.06 | 0.02 (-0.11; 0.15) | 0.06 | 0.13 (1; 20) | 0.73 | 1.23 (23; 344) | 0.21 |  |  |
| Time (%) with impaired cerebral autoregulation (*r*>0.5) | 23.5 | | 21.6 | 1.9 (-7.9; 11.7) | 4.7 | 0.16 (1; 20) | 0.69 | 0.59 (23; 342) | 0.93 |  |  |

Bpm, beats per minute; CI, confidence interval; CRR, cerebrorenal (rSO_2_) ratio; DBP, diastolic blood pressure; dfd, degrees of freedom denominator; dfn, degree of freedom numerator; FTOE, fractional tissue oxygen extraction; HR, heart rate; MABP, mean arterial blood pressure; NIRS, near-infrared spectroscopy; rSO_2,_ regional oxygen saturation; SaO_2_, arterial oxygen saturation;SBP, systolic blood pressure; SE, standard error.

**Table S2.** The effect of prenatal sildenafil versus placebo on neonatal cerebral tissue oxygen saturation (rSO_2_) and fractional tissue oxygen extraction (FTOE) and cerebral autoregulation in severeearly-onset fetal growth restriction, including only infants also receiving renal NIRS measurements (n_sildenafil_ = 6, n_placebo_ = 5).

| **Parameter** | **Mean** | | | **Difference between means (95% CI)** | **SE of difference** | **Treatment** | | **Slope** | | |  |
| --- | --- | --- | --- | --- | --- | --- | --- | --- | --- | --- | --- |
|  | **Placebo** | **Sildenafil** | |  |  | **F (dfn; dfd)** | **p** | **F (dfn; dfd)** | **p** |  |  |
| **NIRS-derived parameters** | | | | | | | | | | | |
| Cerebral rSO_2_ (%) | 79.74 | | 75.30 | 4.44 (-1.08; 9.96) | 2.44 | 3.31 (1; 9) | 0.10 | 0.58 (23; 181) | 0.94 |  |  |
| Cerebral FTOE | 0.14 | | 0.19 | -0.05 (-0.09; -0.001) | 0.02 | 5.29 (1; 9) | 0.047 | 0.61 (23; 138) | 0.92 |  |  |
| **Cerebral autoregulation** | | | | | | | | | | | |
| MABP/cerebral rSO_2_correlation coefficient (*r*) | 0.10 | | 0.03 | 0.06 (-0.12; 0.24) | 0.08 | 0.61 (1; 9) | 0.45 | 1.11 (22; 158) | 0.34 |  |  |
| Time (%) with impaired cerebral autoregulation (*r*>0.5) | 25.8 | | 18.5 | 7.28 (-5.40; 19.96) | 5.61 | 1.69 (1; 9) | 0.23 | 1.10 (23; 158) | 0.35 |  |  |

CI, confidence interval; dfd, degrees of freedom denominator; dfn, degree of freedom numerator; NIRS, near-infrared spectroscopy; SE, standard error.
